# Association of Hyperbilirubinemia with Lipid Profile and Lipid-Related Diseases: A Large Community-Based Cohort Study

**DOI:** 10.3390/jcm15020455

**Published:** 2026-01-07

**Authors:** Borong Yu, Yuhe Liu, Wenqian Wu, Yong Zhou, Dan Han, Yuanwen Chen

**Affiliations:** 1 Department of Gastroenterology, Huadong Hospital, Fudan University, Shanghai 200040, China; bryu23@m.fudan.edu.cn; 2 Department of Public Health and Preventive Medicine, School of Medicine, Tongji University, Shanghai 200331, China; 3 Clinical Research Institute, Shanghai General Hospital, Shanghai Jiao Tong University School of Medicine, Shanghai 200080, Chinayongzhou78214@sjtu.edu.cn (Y.Z.)

**Keywords:** hyperbilirubinemia, hyperlipidemia, bilirubin, lipid profile, cholesterol, cardiovascular disease, metabolic syndrome, atherosclerosis, obesity, antioxidant effect

## Abstract

**Objectives:** Emerging evidence suggests that bilirubin, beyond being a metabolic byproduct, may exert protective effects against metabolic and cardiovascular diseases due to its antioxidant properties. However, its relationship with hyperlipidemia remains unclear. This study investigated the relationship between hyperbilirubinemia and hyperlipidemia in a large, community-based cohort. **Methods:** Data from 8464 participants in the Jidong Community Cohort were analyzed using a cross-sectional design. Hyperbilirubinemia was defined as serum total bilirubin (STB) ≥ 17.1 μmol/L, whereas hyperlipidemia was determined based on a prior diagnosis or elevated lipid profile. **Results:** Of all participants, 31.6% had hyperbilirubinemia and 51.8% had hyperlipidemia. Multivariable logistic regression revealed a significant inverse association between hyperbilirubinemia and hyperlipidemia [odds ratio (OR) = 0.764, 95% confidence interval (CI) = 0.686–0.851]. This association was significant in participants aged <65 years (OR = 0.762, *p* < 0.0001) but not in those aged ≥65 years. Stratified analysis by smoking status further revealed a 29% reduced risk of hyperlipidemia among never-smokers (OR = 0.708, *p* < 0.001), but not among current (OR = 0.831, *p* = 0.087) or former smokers (OR = 0.685, *p* = 0.175). Hyperbilirubinemia was also negatively associated with TC (*p* < 0.0001), TGs (*p* < 0.0001), LDL-C (*p* = 0.0061), very LDL-C (VLDL-C; *p* = 0.0043), and apolipoprotein B (ApoB; *p* < 0.0001) levels, as well as the ApoB/apolipoprotein A1 (ApoA1) ratio (*p* = 0.0003). Restricted cubic spline analysis revealed an inverse relationship of high STB levels with the TC, TG, LDL-C, VLDL-C, and ApoB levels, as well as the ApoB/ApoA1 ratio. Moreover, elevated STB levels were inversely linked to obesity (OR = 0.747, *p* < 0.0001), arterial stenosis (OR = 0.806, *p* = 0.0462), and metabolic syndrome (OR = 0.784, *p* = 0.0008). **Conclusions:** hyperbilirubinemia may be an independent factor protective against hyperlipidemia and related lipid abnormalities; these results provide insights for the prevention and management of lipid-related diseases.

## 1. Introduction

Bilirubin, traditionally considered a biologically inactive byproduct of hemoglobin metabolism, has emerged as a molecule with significant physiological and metabolic roles. It is currently recognized as a potent endogenous antioxidant, protecting cells from oxidative stress and inflammation [1]. In healthy adults, serum total bilirubin (STB) levels typically range from 3.4 to 17.1 μmol/L, and STB ≥ 17.1 μmol/L is considered to indicate hyperbilirubinemia [2]. In addition to antioxidant properties, bilirubin has been identified to play a role as a signaling molecule influencing various metabolic pathways, including those involving peroxisome proliferator-activated receptors (PPARs), fatty acid binding proteins, and apolipoproteins [3]. These findings have spurred interest in bilirubin’s potential protective effects against metabolic and cardiovascular diseases, including obesity, diabetes, and chronic kidney disease (CKD) [4,5,6,7].

Hyperlipidemia, characterized by elevated levels of serum total cholesterol (TC), triglyceride (TG), low-density lipoprotein cholesterol (LDL-C), or reduced levels of high-density lipoprotein cholesterol (HDL-C), is a major risk factor for atherosclerosis and cardiovascular diseases [8]. It is closely linked to unhealthy lifestyle factors such as smoking, alcohol consumption, and physical inactivity; it significantly increases the risks of coronary heart disease, myocardial infarction, and metabolic disorders [9,10]. Although the relationship between hyperlipidemia and cardiovascular risk is well-established, bilirubin’s role in lipid metabolism and its potential protective effects against hyperlipidemia remain underexplored. Some studies have suggested that slight elevations in STB levels are associated with decreases in lipid levels and cardiovascular risk [11,12,13,14,15]; however, others have reported conflicting findings, including no significant association of STB levels with hyperlipidemia, or even indicated a potential role of elevations in STB levels as a risk factor for hyperlipidemia [16,17]. Furthermore, it is well-established that unhealthy lifestyle factors, such as smoking, alcohol consumption, and lack of physical exercise, are closely associated with the risk of dyslipidemia [18]. Notably, smoking has been well-established as an independent factor contributing to an adverse lipid profile, including elevated TG and LDL-C, reduced HDL-C, and a consequently increased risk of hyperlipidemia and cardiovascular events. Importantly, the deleterious impact of smoking on lipid metabolism may persist even after cessation [19,20,21]. Although the relationship between bilirubin and lipids has been investigated, the role of smoking—a significant potential confounder and effect modifier—in this association has not been thoroughly examined within large community-based cohorts.

Beyond its established association with hyperlipidemia, bilirubin has increasingly been linked to a spectrum of cardiovascular and metabolic diseases. Numerous studies have separately investigated the relationship between bilirubin levels and conditions such as obesity, hypertension, arterial stenosis, diabetes, and metabolic syndrome. Regarding obesity, research conducted among middle-aged adults has shown that individuals with obesity or overweight tend to have lower serum bilirubin levels [22,23]. A weight loss exceeding 2% has been associated with a significant increase in bilirubin levels, whereas weight gain is linked to a decrease in bilirubin [24]. Research has shown that elevated serum bilirubin levels may reduce the risk of hypertension by inactivating and inhibiting the synthesis of reactive oxygen species in vascular cells [25,26]. In contrast, a study by Chen et al. further indicated that increased levels of total bilirubin and unconjugated bilirubin are risk factors for hypertension, whereas elevated conjugated bilirubin exhibits the opposite effect [27]. Regarding the association between bilirubin and arterial stenosis and calcification, studies have found that bilirubin deficiency can lead to destabilization of atherosclerotic plaques [28], and low serum bilirubin concentration may serve as a potential risk factor for coronary artery calcification [29,30]. Furthermore, numerous studies have indicated an inverse correlation between serum bilirubin levels and both metabolic syndrome [31,32] and type 2 diabetes [33]. The association between bilirubin levels and nonalcoholic fatty liver disease (NAFLD) remains contentious. While some studies have demonstrated an inverse relationship between elevated bilirubin levels and the prevalence of NAFLD [34,35,36], others have reported no significant association [37,38]. It is noteworthy that there is a current shift towards using the definition of metabolic dysfunction-associated steatotic liver disease (MASLD) [39]. However, as the Jidong cohort in this study was originally designed using the NAFLD criteria, this term will be retained in the subsequent sections for consistency. Additionally, higher serum bilirubin concentrations within the physiological range have been associated with a reduced risk of incidence and progression of chronic kidney disease (CKD) in adults [40,41].

Many of the existing studies in this area have been conducted within specific patient populations or with limited sample sizes, and lack a comprehensive assessment of the association between bilirubin and a complete lipid profile, as well as related diseases, in a large community-based cohort. This study, therefore, aimed to investigate the association between hyperbilirubinemia and lipid subclasses in a large, community-based cohort. Specifically, we evaluated whether elevated STB levels are inversely associated with hyperlipidemia and lipid-related diseases, such as obesity, hypertension, arterial stenosis, and metabolic syndrome. In contrast to previous studies, the present investigation not only examines the overall risk of hyperlipidemia but also employs models such as restricted cubic splines (RCS) to explore dose–response patterns of the associations. It further provides an in-depth analysis of the nonlinear relationships between different lipid components and hyperbilirubinemia, aiming to more comprehensively elucidate the potential protective role of bilirubin in lipid metabolism and related disorders. Moreover, through stratified analyses, we specifically assessed the effect modification by sex, age, and smoking status. Within the same population, we conducted a horizontal comparison of the strength of associations between bilirubin levels and multiple lipid-related metabolic diseases. By leveraging data from the Jidong Community Cohort, we also explored potential mechanisms underlying bilirubin’s protective effects, including its anti-inflammatory, antioxidant, and lipid-regulatory properties. This study provides novel insights into bilirubin’s role in lipid metabolism and its potential implications for lipid-related disease prevention and management.

## 2. Materials and Methods

### 2.1. Data Source and Study Population

The current data source, the Jidong Community Cohort, is a cohort from one of China’s subhealth cohort studies with long-term follow-up. The Jidong community is located in Caofeidian District, Tangshan City, Hebei, China. The participants are primarily employees of the Jidong Oilfield of China National Petroleum Corporation, as well as their families. All participants provided informed consent before study initiation. The study was conducted in accordance with the Helsinki Declaration and approved by the Ethical Committees of the Staff Hospital of Jidong Oilfield of China National Petroleum Corporation.

**Figure 1:**
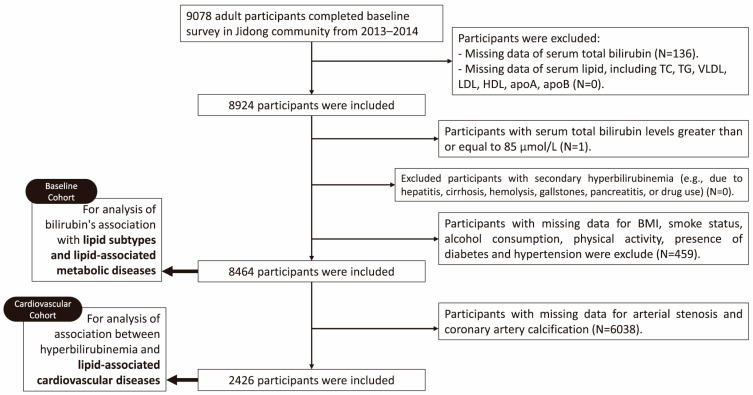
Flow of participant selection.

In the present study, we included data from 9078 participants from the Jidong community, collected from July 2013 to August 2014. The flowchart of participant selection is provided in Figure 1. We initially excluded participants with missing data related to STB, TC, TG, very LDL-C (VLDL-C), LDL-C, HDL-C, apolipoprotein A1 (ApoA1), or apolipoprotein B (ApoB) levels, or other relevant covariates. Moreover, individuals with abnormally elevated STB levels (≥85 μmol/L) were excluded. Specifically, we excluded those with: (1) a clinical diagnosis of hepatitis [42], cirrhosis [43], or other chronic liver diseases [44]; (2) a history of hemolytic anemia [45]; (3) acute conditions such as pancreatitis or biliary obstruction (e.g., gallstones) recorded at baseline [46]; and (4) use of medications known to significantly elevate bilirubin (e.g., certain antivirals, antibiotics) within the preceding 3 months. Consequently, we established a baseline cohort (N = 8464) for investigating the association of STB levels with lipid subclasses and lipid-related metabolic disorders. Subsequently, we identified 2426 participants with complete computed tomography (CT) imaging and vascular ultrasound data to constitute a cardiovascular cohort (N = 2426) to specifically examine the potential relationship of hyperbilirubinemia with arterial stenosis and coronary artery calcification (CAC).

### 2.2. Definition of Hyperbilirubinemia and Hyperlipidemia

Participants with STB ≥ 17.1 μmol/L were classified as patients with hyperbilirubinemia, whereas those with STB < 17.1 μmol/L were considered to have STB levels within the normal range [2,47]. The diagnosis of hyperlipidemia was established based on (1) a previous diagnosis by medical personnel, (2) TC ≥ 5.2 mmol/L, (3) total TG ≥ 1.7 mmol/L, (4) LDL-C ≥ 3.4 mmol/L, or (5) HDL-C < 1.0 mmol/L [48]. Moreover, abnormal apolipoprotein levels were defined as apolipoprotein A1 (ApoA1) < 1.0 g/L, ApoB ≥ 1.2 g/L, or ApoB/ApoA1 ratio ≥ 1.2 [48,49].

### 2.3. Covariates and Data Collection

The following potential covariates were considered in accordance with previous studies: age, sex, body mass index (BMI), smoking status, alcohol consumption, physical activity, presence of diabetes and hypertension, alanine transaminase (ALT), gamma-glutamyl transferase (GGT), uric acid (UA), and serum creatinine (sCr). Participants were interviewed and asked to complete a structured questionnaire on demographic characteristics. Fasting venous blood samples were collected from all participants using standard phlebotomy procedures after an 8 to 12 h fast. The samples were immediately transported to the clinical laboratory of the Staff Hospital of Jidong Oilfield of China National Petroleum Corporation, where serum was separated by centrifugation. All biochemical indicators were then measured using standardized, fully automated analyzers, with strict adherence to quality control protocols throughout the entire process.

Based on their BMI (calculated as weight in kilograms divided by height in meters squared), the participants were categorized as normal weight (<24.0 kg/m^2^), overweight (24.0–28.0 kg/m^2^), and obese (≥28.0 kg/m^2^) [50]. Based on their smoking status, the participants were categorized as never-smokers (individuals who had smoked <100 cigarettes in their lifetime), former smokers (individuals who had smoked ≥100 cigarettes in lifetime, but now had quit for at least 6 months at the time of survey), and current smokers (individuals who had smoked ≥100 cigarettes in lifetime, and were currently smoking) [51]. Regarding alcohol consumption, participants were classified as nondrinkers, daily consumers of less than one standard drink (a beverage containing 10 g of pure alcohol [52]), and daily consumers of more than one standard drink per day. This classification was applied uniformly to all participants, irrespective of sex. Based on physical activity levels, ascertained via structured questionnaire, and the World Health Organization’s recommendations, the participants were categorized as inactive (no planned moderate or vigorous activity), occasional (one or two sessions of <150 min moderate or <75 min vigorous activity per week), and frequently (at least three sessions of ≥150 min moderate or ≥75 min vigorous activity per week) [53].

Blood pressure was measured using an automated digital sphygmomanometer, with the cuff appropriately positioned on the upper right arm at heart level. Hypertension was ascertained based on (1) a previous diagnosis by medical personnel, (2) systolic blood pressure ≥ 140 mmHg, or (3) diastolic blood pressure ≥ 90 mmHg [54]. Diabetes was ascertained based on (1) a previous diagnosis by medical personnel or (2) fasting blood glucose (FBG) ≥ 7.0 mmol/L [55].

### 2.4. Definition of Lipid-Related Diseases

A BMI of ≥28.0 kg/m^2^ was considered to indicate obesity [50]. Arterial stenosis was diagnosed through ultrasound of the bilateral common carotid arteries, internal carotid arteries, or vertebral arteries. Based on the North American Symptomatic Carotid Endarterectomy Trial, the stenosis degree was classified into no or mild stenosis (luminal narrowing < 50%) and severe stenosis (luminal narrowing ≥ 50%) [56]. CAC was assessed using the CAC score, in which calcified plaques in the coronary arteries are measured through CT. All participants provided written informed consent after being fully informed of the potential risks and benefits of the procedure, including the minimal radiation exposure associated with CT. A CAC score of ≥100 was considered to indicate an intermediate to high risk and thus the need for intensified interventions (e.g., statin therapy). In contrast, a CAC score of <100 was regarded as suggesting a low risk [57]. Nonalcoholic fatty liver disease (NAFLD) was diagnosed through ultrasonography in male participants consuming alcohol less than two standard drinks per day and female participants consuming less than one standard drink per day. Diagnosis was based on established sonographic criteria, requiring the presence of at least two of the following key features: (1) hepatic hyperechogenicity, or “bright liver”, compared to the renal cortex or spleen; (2) far-field beam attenuation with deep portion signal loss; and (3) blurring of the intrahepatic vessel borders [58]. All images were acquired and interpreted following a standardized protocol by experienced radiologists. Metabolic syndrome was diagnosed based on a waist circumference of ≥90 cm in men and ≥80 cm in women, along with the presence of at least two of the following four abnormal criteria: (1) TG abnormalities (TG ≥ 1.7 mmol/L), (2) blood pressure abnormalities (SBP ≥ 130 mmHg, diastolic blood pressure ≥ 85 mmHg, hypertension history, or antihypertensive medication use), (3) HDL-C abnormalities (HDL-C < 1.03 mmol/L in men or HDL-C < 1.29 mmol/L in women), and (4) blood glucose abnormalities (FBG ≥ 5.6 mmol/L or type 2 diabetes history) [59]. CKD was diagnosed based on an estimated glomerular filtration rate (eGFR) of <60 mL/(min·1.73 m^2^) [60]; the eGFR was calculated using the sCr-based 2021 CKD-EPI equation:

eGFR = 142 × min(sCr/κ, 1)^α^ × max(sCr/κ, 1)^−1.200^ × 0.9938^age^ × [1.012 if female],

where κ = 0.7 for females and 0.9 for males; α = −0.241 for females and −0.302 for males; and sCr concentrations are expressed in mg/dL [61].

### 2.5. Statistical Analysis

Categorical variables are expressed as numbers (percentages); continuous variables with a normal distribution are presented as mean ± standard deviation (SD), whereas those with an abnormal distribution are expressed as median [Q1, Q3]. All statistical analyses were conducted using SAS (version 9.4; SAS Institute Inc., Cary, NC, USA) and R (version 4.5.0; R Foundation for Statistical Computing, Vienna, Austria). Two-sided *p* < 0.05 was considered to indicate statistical significance.

We assessed data normality using the Kolmogorov–Smirnov test (for sample sizes ≥ 50) in both hyperbilirubinemia and nonhyperbilirubinemia groups; however, both groups demonstrated significant deviations from normality (*p* < 0.05). Consequently, we employed the weighted chi-square or Mann–Whitney U test rather than the t test to compare basic characteristics between the two aforementioned groups. Multivariable logistic regression models were employed to calculate the odds ratios (ORs), 95% confidence intervals (CIs), and *p* values for the association of STB levels with various dependent variables. Moreover, subgroup analysis was performed according to age and sex. RCS models were used to assess the nonlinear association of STB levels with the lipid subclasses. Knot configurations were established based on sample distribution and clinical guidelines, which included the 10th, 50th, and 90th percentiles, as well as the thresholds of 17.1 and 34.2 μmol/L. Both predicted values (covariate-adjusted expected values) and ORs (derived from covariate-adjusted logistic regression) were visualized in the plotted curves. The multicollinearity among variables was assessed using the variance inflation factor (VIF). All adjusted VIF values [GVIF (1/(2 × Df))] ranged between 1.01 and 1.67, considerably lower than the threshold of 5, indicating no significant multicollinearity.

We employed the R package mice (version 3.16.0) to perform multiple imputation for handling missing data in partial variables. In total, 20 imputed datasets (m = 20) with 10 iterations each were generated, with a random seed (2024) set to ensure reproducibility. Continuous variables were imputed using predictive mean matching, whereas binary variables were imputed through logistic regression. The predictor matrix was constructed with the following criteria: a minimum correlation coefficient between variables of 0.1 and a usable cases proportion of at least 5% (minpuc = 0.05).

## 3. Results

### 3.1. Association of Hyperbilirubinemia with Lipid Subclasses

#### 3.1.1. Baseline Characteristics of Our Baseline Cohort

Of the 9078 participants enrolled in the Jidong cohort over 2013–2014, 8464 participants who met our inclusion and exclusion criteria were included in our baseline cohort and analyzed (Figure 1). Table 1 lists the baseline characteristics of the included participants, grouped by the diagnosis of hyperbilirubinemia. The median [Q1, Q3] age of the included participants was 41 [31, 52] years; 4382 (51.77%) participants were male, 4383 (51.78%) had hyperlipidemia, and 2672 (31.57%) had hyperbilirubinemia. In general, sex, BMI, alcohol consumption, smoke status, hypertension, and levels of TC, LDL-C, HDL-C, ApoA1, ApoB, ALT, aspartate aminotransferase (AST), GGT, sCr, and UA demonstrated significant differences between the hyperbilirubinemia and nonhyperbilirubinemia groups (all *p* < 0.05).

#### 3.1.2. Association of Hyperbilirubinemia with Hyperlipidemia

To analyze the relationship between hyperbilirubinemia and hyperlipidemia, multivariable logistic models were established, and stratified subgroup analysis was performed (Table 2). Stratified analyses revealed population-specific associations between hyperbilirubinemia and hyperlipidemia. In fully adjusted models, the total population demonstrated a 24% risk reduction associated with hyperbilirubinemia (OR = 0.764, *p* < 0.001). After age stratification, a significant 24% risk reduction was associated with hyperbilirubinemia in participants aged <65 years (*p* < 0.001); however, no such significant association was noted in those aged ≥65 years. After sex stratification, hyperbilirubinemia demonstrated protective effects in both men (OR = 0.823, *p* = 0.005) and women (OR = 0.665, *p* < 0.001); nevertheless, risk reduction was greater in women (33.5% vs. 17.7%). Further stratification by smoking status showed a distinct pattern. Among never-smokers, hyperbilirubinemia exhibited a significant protective effect, being associated with a 29% reduced risk of hyperlipidemia (OR = 0.708, *p* < 0.001). However, no statistically significant associations were observed in either current smokers (OR = 0.831, *p* = 0.087) or former smokers (OR = 0.685, *p* = 0.175) after full adjustment.

It is worth noting that, to examine the potential influence of lipid-lowering drug use, we performed a sensitivity analysis by excluding all participants who reported using statins or fibrates (n = 108, Table 1). The results from the fully adjusted model (Model 3) remained largely unchanged. The inverse association between hyperbilirubinemia and hyperlipidemia persisted with a similar effect size (OR = 0.729 95% CI: 0.653–0.815, *p* < 0.001).

#### 3.1.3. Logistic Regression Analysis of Association of Hyperbilirubinemia with Lipid Subclasses

Multivariate analysis revealed significant associations of hyperbilirubinemia with various lipid subclasses (Table 3). In fully adjusted models, elevated TC (≥5.2 mmol/L), TG (≥1.7 mmol/L), LDL-C (≥3.4 mmol/L), and ApoB (≥1.2 g/L) levels were significantly associated with a lower hyperbilirubinemia risk. Similar protective associations were observed for VLDL-C levels (≥0.78 mmol/L) and the ApoB/ApoA1 ratio (≥1.2). The associations of hyperbilirubinemia with HDL-C (<1.0 mmol/L) and ApoA1 (<1.0 g/L) levels became nonsignificant after full adjustment.

#### 3.1.4. RCS Analysis of STB Levels and Lipid Subclasses

RCS models were employed to evaluate the nonlinear relationships of STB levels with various lipid subclasses. To mitigate the influence of extreme outliers, the median STB level was selected as the reference point (OR = 1). The results demonstrated that increasing STB levels were inversely associated with TC, TG, LDL-C, and ApoB levels, as well as the ApoB/ApoA1 ratio; however, no such significant associations were noted for VLDL-C, HDL-C, and ApoA1 levels.

Hyperbilirubinemia significantly reduced serum TC, TG, LDL-C, and ApoB levels, as well as the ApoB/ApoA1 ratio. When STB levels increased from 17.1 to 34.2 μmol/L, the predicted TC levels decreased from 4.17 to 3.96 mmol/L. In the TC ≥ 5.2 mmol/L subgroup, the OR (95% CI) decreased from 0.83 (0.73–0.94) to 0.49 (0.30–0.79). Similarly, in the TG ≥ 1.7 mmol/L subgroup, the predicted TG levels decreased from 1.15 to 1.05 mmol/L, with corresponding reductions in OR (95% CI) from 0.95 (0.85–1.06) to 0.61 (0.49–0.78). In the LDL-C ≥ 3.4 mmol/L subgroup, LDL-C levels exhibited a dose-dependent decrease, with OR (95% CI) declining from 0.78 (0.65–0.94) to 0.49 (0.30–0.79). At 34.2 μmol/L STB, both ApoB levels (OR = 0.44, 95% CI: 0.33–0.60) and the ApoB/ApoA1 ratio (OR = 0.39, 95% CI: 0.26–0.59) demonstrated significant reductions.

**Figure 2:**
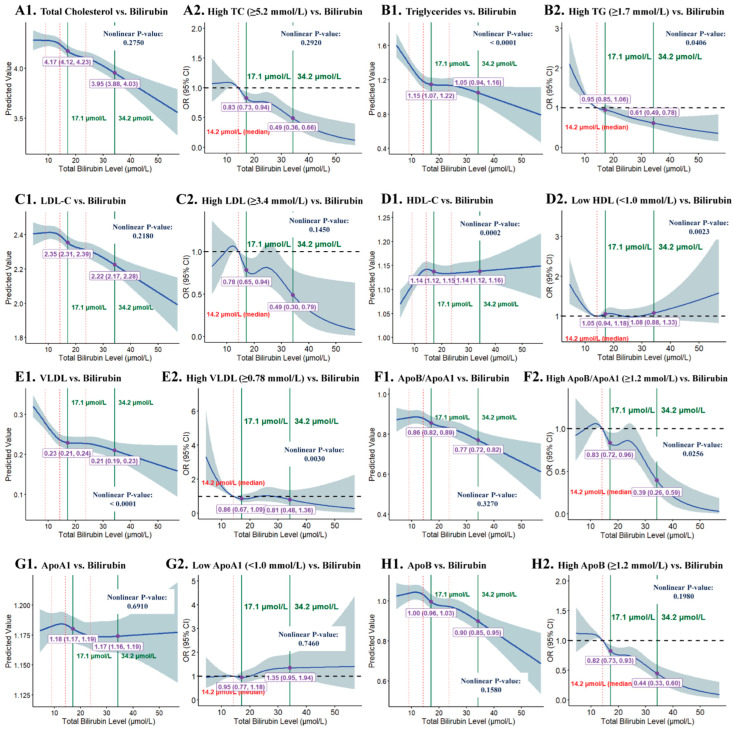
RCS plots for association of STB with different lipid subclasses. (a) Knot configurations were defined by the 10th, 50th, and 90th percentiles, with additional thresholds set at 17.1 and 34.2 μmol/L. (b) Models were adjusted for age, sex, smoking status, alcohol consumption, physical activity, BMI, diabetes, hypertension, ALT, GGT, UA, and sCr. (c) Blue solid lines represent predicted values and ORs, whereas shaded gray areas indicate the 95% CIs. Red dashed lines indicate the 10th, 50th, and 90th percentiles of the distribution. Green solid lines indicate the positions of total bilirubin levels at 17.1 μmol/L and 34.2 μmol/L, respectively. Small dots represent the intersections of the green solid lines and the blue solid line, indicating the positions of total bilirubin levels at 17.1 μmol/L and 34.2 μmol/L on the blue solid line.

In contrast, no significant associations were observed between STB levels and HDL-C, VLDL-C, or ApoA1 levels. VLDL-C levels demonstrated an initial rapid decline at STB ≤ 17.1 μmol/L, followed by stabilization; HDL-C and ApoA1 levels exhibited only marginal increases at STB > 17.1 μmol/L. All three parameters demonstrated nonsignificant changes in OR at STB > 17.1 μmol/L with 95% CIs overlapping. Nevertheless, we observed that as STB levels increased, HDL-C and ApoA1 levels increased gradually, whereas VLDL-C levels decreased (Figure 2).

### 3.2. Association of Hyperbilirubinemia with Lipid-Related Diseases

#### 3.2.1. Baseline Characteristics of Our Cardiovascular Cohort

Our cardiovascular cohort comprised 2426 participants, of whom 31.74% had hyperbilirubinemia and 59.05% had hyperlipidemia (Table 4). Compared with our baseline cohort, this cohort had a higher mean age and BMI. The hyperbilirubinemia group also exhibited significant sex differences, with a notably higher proportion of males than females (68.57%). Moreover, without covariate adjustment, hypertension and NAFLD prevalence demonstrated statistically significant differences between the hyperbilirubinemia and nonhyperbilirubinemia groups. Nevertheless, the baseline analysis revealed no significant differences between the two groups in terms of age, diabetes, hyperlipidemia, arterial stenosis, CAC, obesity, metabolic syndrome, or CKD (all *p* > 0.05).

#### 3.2.2. Logistic Regression Analysis of Association of Hyperbilirubinemia with Lipid-Related Diseases

After adjustments for covariates, multivariate regression analysis revealed significant variations in the association of hyperbilirubinemia with various lipid-related diseases. Among cardiovascular diseases, arterial stenosis showed a significant inverse association with hyperbilirubinemia in the fully adjusted model (Model 3: OR = 0.806, *p* = 0.046), whereas CAC demonstrated no significant association with hyperbilirubinemia across all models (*p* > 0.05). Among metabolic diseases, obesity (Model 3: OR = 0.747, *p* < 0.0001) and metabolic syndrome (Model 3: OR = 0.784, *p* = 0.0008) were significantly and inversely associated with hyperbilirubinemia. Hypertension showed no significant association with hyperbilirubinemia in the adjusted model; moreover, CKD and diabetes demonstrated no significant association with hyperbilirubinemia in any model. NAFLD demonstrated an inverse association with hyperbilirubinemia in the partially adjusted model (Model 2: OR = 0.829, *p* = 0.0003) but lost significance after full adjustment (*p* = 0.3265) (Table 5).

## 4. Discussion

In this study, we investigated the relationship of hyperbilirubinemia with serum lipid subclasses and lipid-related diseases in a large cohort from the Jidong community. Our findings revealed a significant inverse association between hyperbilirubinemia and hyperlipidemia, particularly in participants aged <65 years. Stratified analysis by smoking status further revealed a 29% reduced risk of hyperlipidemia among never-smokers (OR = 0.708, *p* < 0.001), but not among current (OR = 0.831, *p* = 0.087) or former smokers (OR = 0.685, *p* = 0.175). Hyperbilirubinemia was also associated with lower TC, TG, LDL-C, and ApoB levels, as well as the ApoB/ApoA1 ratio. Furthermore, hyperbilirubinemia was significantly associated with reduced risks of obesity, arterial stenosis, and metabolic syndrome but not with CAC, hypertension, diabetes, NAFLD, or CKD. Thus, hyperbilirubinemia may be an independent protective factor against hyperlipidemia and certain lipid-related diseases.

Our study extends the existing evidence in several key aspects. First, it reveals, through a comprehensive analysis, the impact pattern of hyperbilirubinemia on various lipoprotein and apolipoprotein components. Second, it identifies an age-specific nature of this association, a dimension that has received limited emphasis in previous research, while simultaneously conducting stratified analyses to examine the modifying effect of smoking—a key factor—on the relationship between bilirubin and lipids. Finally, the use of a uniform cohort design reduces bias arising from population heterogeneity across different population.

### 4.1. Comparison with Previous Studies

Our findings align with previous research suggesting that STB has protective effects against lipid abnormalities and cardiovascular diseases. For instance, Kawamoto et al. reported a negative correlation between STB levels and carotid atherosclerosis, highlighting bilirubin’s potential role in mitigating cardiovascular risk factors [15]. Similarly, Hou et al. demonstrated that elevated STB levels are inversely associated with cardiovascular risk; however, the authors did not establish a causal relationship between elevated STB levels and diabetes risk [62]. In contrast, Wei et al. indicated that high STB levels may reduce type 2 diabetes risk [6]. Although we did not note a significant association between hyperbilirubinemia and diabetes, our results support the broader hypothesis that STB exerts protective effects on metabolic health.

The relationship between STB and lipid levels remains a topic of debate. For instance, Khoei et al. have shown that mildly elevated STB levels are associated with lower TG levels, particularly in men [13]. In contrast, Oda reported that in men, STB levels are positively correlated with TG levels and negatively correlated with HDL-C levels [16]. Moreover, Zhang et al. reported no significant association between STB and TG levels in a Chinese population [17]. These discrepancies may be attributable to differences in study populations and methodologies, as well as in the categorization of STB into direct bilirubin (DBil) and indirect bilirubin (IBil). For instance, Fu et al. observed a negative correlation between DBil and TC; in contrast, Bai et al. suggested that DBil acts as a protective factor against TC and TG elevation, whereas IBil may be a risk factor for elevated TC levels [63,64]. In the current study, we focused on STB, and our results demonstrating a consistent inverse relationship between hyperbilirubinemia and lipid levels in a large Chinese cohort add to this body of evidence.

### 4.2. Observations and Possible Interpretations

The stratified analyses in this study revealed that the protective effect of hyperbilirubinemia against hyperlipidemia was significant in individuals aged <65 years but was not observed in those aged ≥65 years. This finding differs from the research by Vitek et al., who observed improved body fat parameters and lipid profiles in patients with Gilbert’s syndrome (GS) even among individuals aged ≥35 years [65]. We speculate that the discrepancy may be attributed to the following reasons. On one hand, Vitek et al. focused on patients with Gilbert’s syndrome, a condition characterized by chronic, mild elevation of unconjugated bilirubin, whereas our study involved a community-based general elderly population. On the other hand, and more critically, the elderly community population typically exhibits an accumulation of age-related comorbidities, such as declining renal function, polypharmacy, and progressive vascular stiffness. These potent pro-atherogenic factors may mask or outweigh the relatively modest protective effect of bilirubin.

The association between hyperbilirubinemia and hyperlipidemia varied across different smoking status groups. After full adjustment, a protective association remained robust and consistent only in never-smokers, was attenuated in current smokers, and was absent in former smokers. This indicates that smoking status serves as a key effect modifier. The attenuation of the association in current smokers may be attributed to the intense pro-inflammatory and pro-oxidative state induced by smoking [66], which could counteract or obscure the antioxidant and anti-inflammatory benefits of bilirubin. The lack of association observed in former smokers, in addition to the reasons mentioned above, might also be due to the relatively small sample size and limited statistical power in this subgroup, or to residual confounding from long-term metabolic alterations following smoking cessation.

NAFLD is closely associated with dyslipidemia and may involve mild alterations in liver enzymes or biliary excretion function, which could influence bilirubin levels. In our investigation of lipid-related disorders, the association between NAFLD and hyperbilirubinemia was significant after adjusting for age and sex, but became non-significant after further adjustment for metabolic factors such as BMI, diabetes, and hypertension. In contrast, the inverse association between hyperbilirubinemia and blood lipids remained independent. As noted in the Introduction, the relationship between NAFLD and bilirubin levels remains controversial in the literature. Our findings suggest that there is no statistically significant correlation between NAFLD and bilirubin levels, a relationship likely driven mainly by shared metabolic disturbances. The protective association of hyperbilirubinemia appears to be, to some extent, independent of the presence of NAFLD.

Another intriguing secondary observation in our study was that individuals with hyperbilirubinemia had slightly higher serum creatinine levels compared to those without, although both values remained within the normal reference range and were accompanied by comparable eGFR. This suggests that the observed difference in sCr is unlikely to reflect clinically significant renal impairment. The mechanism behind this subtle divergence is not entirely clear but may be related to the complex physiological interplay between bilirubin metabolism and renal hemodynamics or tubular function.

### 4.3. Potential Mechanisms

The protective effects of STB on lipid metabolism and lipid-related diseases may be explained by several mechanisms. First, bilirubin’s anti-inflammatory properties may play a critical role. Chronic low-grade inflammation is a key contributor to hyperlipidemia and atherosclerosis [67]. Bilirubin can suppress inflammatory biomarkers, such as C-reactive protein, which may indirectly improve lipid metabolism and reduce lipid levels [68]. Second, bilirubin’s antioxidant properties are well-documented and may contribute to its lipid-lowering effects. Oxidative stress is a major driver of atherosclerosis and other lipid-related diseases [69]. Bilirubin, as a potent endogenous antioxidant, can inhibit lipid peroxidation, thereby protecting lipids from oxidative damage and reducing serum lipid levels [70]. The oxidation–reduction cycle of bilirubin and biliverdin, mediated by biliverdin reductase, amplifies bilirubin’s antioxidant capacity, potentially enhancing its protective effects against lipid peroxidation [71,72]. Moreover, bilirubin is lipophilic, interacting directly with lipids and further preventing oxidative damage [73]. Furthermore, unconjugated bilirubin can inhibit LDL-C oxidation, reducing atherosclerosis risk [74].

Third, a principal mechanism underlying the lipid-improving effect of bilirubin involves the activation of the nuclear receptor peroxisome proliferator-activated receptor alpha (PPARα) [75]. PPARα, strongly expressed in the liver, is central to fatty acid oxidation, TG metabolism, and lipoprotein regulation [76]. Research indicates that unconjugated bilirubin (UCB) can function as an endogenous, selective ligand for PPARα. Following its uptake into hepatocytes via specific transporters, UCB directly interacts with PPARα to enhance its transcriptional activity. This interaction modulates the recruitment of coregulator proteins to the receptor complex, thereby altering gene expression and promoting the transcription of a suite of enzymes involved in lipid catabolism [75]. Importantly, this beneficial metabolic action is specifically attributable to UCB, which can readily enter cells and engage nuclear receptors. In contrast, the hydrophilic nature of conjugated bilirubin precludes it from mediating this specific effect. This distinction provides a critical interpretive lens for our findings: whereas an elevation in total bilirubin may result from various pre-hepatic or hepatic pathologies not associated with metabolic benefit (e.g., hemolysis or cholestasis), the inverse correlation between bilirubin and blood lipid levels observed in this study likely reflects a state of mild, physiological elevation of UCB rather than pathological hyperbilirubinemia. It is this specific state that activates the PPARα pathway, conferring the observed metabolic improvement in lipid profiles. This mechanistic hypothesis is corroborated by the work of Gordon et al., who demonstrated that bilirubin influences lipid homeostasis by reshaping the PPARα coregulator profile, offering a plausible explanation for its lipid-lowering effects [77]. This mechanism may explain the observed associations of hyperbilirubinemia with lower TC, TG, LDL-C, and VLDL-C levels in the current study.

### 4.4. Implications and Future Directions

Our findings highlight the potential clinical significance of STB levels as a biomarker for lipid metabolism and lipid-related diseases. Elevated STB levels may have an inverse influence on hyperlipidemia, obesity, arterial stenosis, and metabolic syndrome. Thus, STB could be explored as a therapeutic target for preventing and managing lipid-related disorders. For instance, interventions to mildly increase STB levels, such as pharmacological modulation of heme oxygenase-1, could be investigated for their potential benefits in lipid metabolism and cardiovascular health.

### 4.5. Limitations

Future studies should also address the following limitations of the current study. First, the cross-sectional design of our study precludes the establishment of causal relationships between hyperbilirubinemia and lipid subclasses. Longitudinal studies are needed to confirm the temporal relationship between STB levels and lipid metabolism. Second, we did not differentiate between DBil and IBil, which may have distinct effects on lipid subclasses. Additional studies are required to explore the differential roles of DBil and IBil in lipid metabolism and lipid-related diseases. Third, the generalizability of our findings may be limited to the northern Chinese population. Studies including more diverse populations are needed to validate our results and assess their applicability to other ethnic groups.

Fourth, information on lipid-lowering medications in this study was limited. During the design of the cohort and data collection, statins and fibrates were reported in a combined manner, which precluded separate assessment of their independent effects on the observed associations or their potential interactions with bilirubin. This is particularly relevant given the possible competition between fibrates and bilirubin via the PPARα pathway.

Fifth, this study lacks direct biomarker evidence regarding the potential mechanistic pathways of bilirubin. Baseline data did not include inflammatory markers such as C-reactive protein, white blood cell count, or fibrinogen, nor oxidative stress markers such as malondialdehyde or glutathione peroxidase. Consequently, our discussion of the underlying mechanisms represents a reasonable scientific inference rather than a direct validation based on the data from this study. Future prospective or case–control studies should prioritize the inclusion of these mechanistic biomarkers.

Sixth, while we excluded known secondary causes of elevated bilirubin such as liver disease and hemolysis, the definition of hyperbilirubinemia in this study (STB ≥ 17.1 μmol/L) is phenomenological. In a community-based population, it was not feasible to perform genetic testing to clearly distinguish Gilbert syndrome (hereditary unconjugated hyperbilirubinemia) from other unexplained forms of mild hyperbilirubinemia. Consequently, the observed associations likely predominantly reflect characteristics of a “benign” hyperbilirubinemia group in which the Gilbert syndrome phenotype is predominant. Future mechanistic studies should incorporate genotyping to clarify this distinction.

## 5. Conclusions

Our results indicate that hyperbilirubinemia is inversely associated with hyperlipidemia and certain lipid-related diseases (i.e., obesity, arterial stenosis, and metabolic syndrome). These findings support the hypothesis that STB plays an inverse role in lipid metabolism and cardiovascular health; therefore, it may be a therapeutic target for preventing and managing lipid-related diseases. The potential mechanisms underlying these effects involve bilirubin’s anti-inflammatory and antioxidant properties, as well as its PPARα-mediated regulation of lipid metabolism. Further research is warranted to elucidate the causal pathways and therapeutic potential of STB in the treatment of lipid-related disorders.

Our results indicate that, within a large community-based population, hyperbilirubinemia is independently associated with a reduced risk of hyperlipidemia and of specific lipid-related disorders, including obesity, arterial stenosis, and metabolic syndrome. These findings support and lend specificity to the hypothesis that bilirubin may serve as a potential protective factor for cardiometabolic health. The underlying mechanisms likely involve bilirubin’s antioxidant and anti-inflammatory properties, as well as its regulatory effects on lipid metabolism. From a clinical perspective, our study suggests that incorporating bilirubin into comprehensive risk assessment models could facilitate a more refined identification of high-risk individuals. Future research should focus on elucidating the specific roles of different bilirubin components and exploring the possibility of safely modulating bilirubin levels to prevent dyslipidemia.

## Figures and Tables

**Table 1:** Baseline characteristics of participants in our baseline cohort grouped by presence of hyperbilirubinemia.

Characteristic		Total	Nonhyperbilirubinemia	Hyperbilirubinemia	*p*
		*n* = 8464	*n* = 5792	*n* = 2672	
Age (years, median [Q1, Q3])		41 [31, 52]	41 [31, 52]	41 [31, 53]	0.6800
Sex, *n* (%)					<0.0001
	Male	4382 (51.77)	2534 (43.75)	1848 (69.16)	
	Female	4082 (48.23)	3258 (56.25)	824 (30.84)	
Physical Activity, *n* (%)					0.0826
	Inactive	2704 (31.95)	1893 (32.68)	811 (30.35)	
	Occasional	1158 (13.68)	793 (13.69)	365 (13.66)	
	Frequently	4602 (54.37)	3106 (53.63)	1496 (55.99)	
Alcohol Consumption, *n* (%)					<0.0001
	Never	5658 (66.85)	4120 (71.13)	1538 (57.56)	
	<1 standard drink	1255 (14.83)	740 (12.78)	515 (19.27)	
	≥1 standard drink	1551 (18.32)	923 (16.09)	619 (23.17)	
Smoke Status, *n* (%)					<0.0001
	Never	5983 (70.69)	4247 (73.33)	1736 (64.97)	
	Current	2159 (25.51)	1370 (23.65)	789 (29.53)	
	Former	322 (3.80)	175 (3.02)	147 (5.50)	
Presence of Hypertension, *n* (%)					<0.0001
	No	5768 (68.15)	4045 (69.84)	1723 (64.48)	
	Yes	2696 (31.85)	1747 (30.16)	949 (35.52)	
Presence of Diabetes, *n* (%)					0.1587
	No	7885 (93.16)	5411 (93.42)	2474 (92.59)	
	Yes	579 (6.84)	381 (6.58)	198 (7.41)	
Presence of Hyperlipidemia, *n* (%)					0.8637
	No	4081 (48.22)	2789 (48.15)	1292 (48.35)	
	Yes	4383 (51.78)	3003 (51.85)	1380 (51.65)	
BMI (kg/m^2^, median [Q1, Q3])		24 [22, 27]	24 [22, 27]	24 [22, 27]	0.3155
BMI, *n* (%)					0.1457
	Normal	3941 (46.56)	2728 (47.10)	1213 (45.40)	
	Overweight	3152 (36.92)	2098 (36.22)	1027 (38.44)	
	Obese	1398 (16.52)	966 (16.68)	432 (16.17)	
Laboratory Features (median [Q1, Q3])					
Lipid Subclasses					
	VLDL (mmol/L)	0.25 [0.17, 0.38]	0.25 [0.17, 0.38]	0.24 [0.17, 0.37]	0.0804
	LDL-C (mmol/L)	2.44 [2.05, 2.87]	2.46 [2.06, 2.89]	2.39 [2.02, 2.82]	<0.0001
	HDL-C (mmol/L)	1.16 [1.00, 1.35]	1.17 [1.01, 1.36]	1.13 [0.98, 1.33]	<0.0001
	ApoA1 (g/L)	1.22 [1.12, 1.33]	1.23 [1.13, 1.34]	1.21 [1.11, 1.32]	<0.0001
	ApoB (g/L)	0.96 [0.82, 1.13]	0.97 [0.82, 1.15]	0.95 [0.81 1.09]	<0.0001
	TC (mmol/L)	4.38 [3.83, 5.00]	4.43 [3.87, 5.05]	4.28 [3.74, 4.90]	<0.0001
	TGs (mmol/L)	1.24 [0.85, 1.88]	1.24 [0.85, 1.90]	1.22 [0.85, 1.84]	0.0798
Other Parameters					
	ALT (U/L)	18.6 [13.1, 28.5]	18.1 [12.8, 27.2]	20.1 [14.1, 30.7]	<0.0001
	AST (U/L) *	21.0 [18.0, 26.0]	21.0 [18.0, 25.0]	22.0 [18.0, 27.0]	<0.0001
	GGT (U/L)	19.6 [13.3, 32.0]	18.6 [13.0, 30.0]	22.0 [14.7, 35.8]	<0.0001
	ALP (U/L)	65.0 [54.0, 79.0]	65.0 [54.0, 79.0]	66.0 [55.0, 78.0]	0.2877
	sCr (µmol/L)	76.1 [66.8, 85.9]	73.8 [65.6, 84.2]	80.6 [70.6, 88.6]	<0.0001
	UA (µmol/L)	289 [231, 354]	280 [226, 345]	307 [247, 372]	<0.0001
	eGFR (mL/(min·1.73 m^2^))	97.5 [86.4, 108.4]	97.5 [86.0, 108.4]	97.3 [87.2, 108.3]	0.5228
	FBG (mmol/L)	5.0 [4.7, 5.4]	5.0 [4.7, 5.4]	5.0 [4.7, 5.4]	0.9566
Drug Use					
Statins or Fibrate Use, *n* (%)					0.6625
	No	8356 (98.72)	5716 (98.96)	2640 (98.80)	
	Yes	108 (1.28)	76 (1.31)	32 (1.20)	

Notes: BMI, body mass index; VLDL, very low-density lipoprotein cholesterol; LDL-C, low-density lipoprotein cholesterol; HDL-C, high-density lipoprotein cholesterol; Apo, apolipoprotein; ALT, alanine transaminase; GGT, gamma-glutamyl transpeptidase; sCr, serum creatinine; UA, uric acid; eGFR, estimated glomerular filtration rate; FBG, fasting blood glucose. * AST is the variable after multiple imputation, with an original missing data rate of 25.0%.

**Table 2:** Multivariable logistic regression model and subgroup analyses evaluating association of hyperbilirubinemia with hyperlipidemia.

Subgroup		Model 1	Model 2	Model 3
		OR (95% CI), *p*	OR (95% CI), *p*	OR (95% CI), *p*
		Reference	Reference	Reference
Total participants		0.992 (0.905, 1.087), 0.8636	0.710 (0.641, 0.785), <0.0001	0.764 (0.686, 0.851), <0.0001
Age (years)				
	<65	1.008 (0.917, 1.107), 0.8702	0.704 (0.634, 0.781), <0.0001	0.762 (0.682, 0.851), <0.0001
	≥65	0.729 (0.474, 1.120), 0.1489	0.718 (0.463, 1.115), 0.1401	0.755 (0.475, 1.198), 0.2326
Sex				
	Male	0.752 (0.663, 0.852), <0.0001	0.749 (0.661, 0.849), <0.0001	0.823 (0.720, 0.942), 0.0046
	Female	0.674 (0.572, 0.794), <0.0001	0.644 (0.541, 0.767), <0.0001	0.665 (0.554, 0.799), <0.0001
Smoking status				
	Never	0.985 (0.856, 1.072), 0.4530	0.697 (0.615, 0.791), <0.0001	0.708 (0.618, 0.811), <0.0001
	Current	0.798 (0.661, 0.964), 0.0191	0.784 (0.649, 0.948), 0.0122	0.831 (0.672, 1.027), 0.0866
	Former	0.776 (0.483, 1.246), 0.2936	0.769 (0.478, 1.237), 0.2789	0.685 (0.397, 1.183), 0.1750

Notes: “Reference” indicates that the nonhyperbilirubinemia group was the reference category (OR = 1) for comparison. Model 1: no covariates were adjusted. Model 2: age and sex were adjusted. Model 3: age, sex, smoking status, alcohol consumption, physical activity, BMI, diabetes, hypertension, ALT, GGT, UA, and sCr were adjusted.

**Table 3:** Multivariable logistic regression model analysis evaluating association of hyperbilirubinemia with different lipid subclasses.

Subclass	Model 1	Model 2	Model 3
	OR (95% CI), *p*	OR (95% CI), *p*	OR (95% CI), *p*
	Reference	Reference	Reference
Sterol Lipid			
TC ≥ 5.2	0.786 (0.697, 0.887), <0.0001	0.741 (0.653, 0.842), <0.0001	0.730 (0.640, 0.833), <0.0001
Neutral Lipid			
TG ≥ 1.7	0.920 (0.833, 1.018), 0.1056	0.697 (0.627, 0.776), <0.0001	0.715 (0.634, 0.806), <0.0001
Lipoproteins			
LDL-C ≥ 3.4	0.786 (0.655, 0.944), 0.0099	0.752 (0.622, 0.909), 0.0032	0.763 (0.628, 0.925), 0.0061
HDL-C < 1.0	1.208 (1.088, 1.342), 0.0004	0.875 (0.783, 0.978), 0.0184	0.955 (0.851, 1.072), 0.4349
VLDL-C ≥ 0.78	0.902 (0.720, 1.130), 0.3687	0.680 (0.540, 0.857), 0.0011	0.696 (0.542, 0.893), 0.0043
Apolipoproteins			
ApoA1 < 1.0	1.386 (1.134, 1.694), 0.0015	1.048 (0.852, 1.288), 0.6581	1.134 (0.918, 1.401), 0.2437
ApoB ≥ 1.2	0.808 (0.721, 0.905), 0.0002	0.684 (0.606, 0.773), <0.0001	0.698 (0.615, 0.792), <0.0001
ApoB/ApoA1 ratio ≥ 1.2	0.883 (0.773, 1.007), 0.0641	0.729 (0.634, 0.837), <0.0001	0.765 (0.662, 0.883), 0.0003

Notes: “Reference” indicates that the nonhyperbilirubinemia group was the reference category (OR = 1) for comparison. Model 1: no covariates were adjusted. Model 2: age and sex were adjusted. Model 3: age, sex, smoking status, alcohol consumption, physical activity, BMI, diabetes, hypertension, ALT, GGT, UA, and sCr were adjusted.

**Table 4:** Baseline characteristics of participants in our cardiovascular cohort grouped by presence of hyperbilirubinemia.

Characteristic	Total	Nonhyperbilirubinemia	Hyperbilirubinemia	*p*
	*n* = 2426	*n* = 1656	*n* = 770	
Age (years, median [Q1, Q3])	57 [49, 61]	57 [49, 61]	57 [49, 61]	0.7032
Sex (male), *n* (%)	1200 (49.46)	672 (40.58)	528 (68.57)	<0.0001
Presence of Hypertension, *n* (%)	1196 (49.30)	792 (47.83)	404 (52.47)	0.0333
Presence of Diabetes, *n* (%)	323 (13.31)	211 (12.74)	112 (14.55)	0.2235
Presence of Hyperlipidemia, *n* (%)	1494 (61.58)	1039 (62.74)	455 (59.09)	0.0853
Presence of Arterial Stenosis, *n* (%)	636 (26.22)	432 (26.09)	204 (26.49)	0.8322
Presence of CAC, *n* (%)	178 (7.34)	114 (6.88)	64 (8.31)	0.2094
BMI (kg/m^2^, median [Q1, Q3])	25 [23, 27]	25 [23, 27]	25 [23, 27]	0.0937
Presence of Obesity, *n* (%)	433 (17.85)	300 (18.12)	133 (17.27)	0.6137
Presence of Metabolic Syndrome, *n* (%)	1067 (43.98)	743 (44.87)	324 (42.08)	0.1976
Presence of NAFLD *, *n* (%)	1381 (56.92)	915 (55.25)	466 (60.52)	0.0167
Presence of CKD, *n* (%)	61 (2.51)	46 (2.78)	15 (1.95)	0.2244

* NAFLD is the variable after multiple imputation, with an original missing data rate of 13.8%.

**Table 5:** Multivariable logistic regression model analysis evaluating association of hyperbilirubinemia with different lipid-related diseases.

Disease	Model 1	Model 2	Model 3
	OR (95% CI), *p*	OR (95% CI), *p*	OR (95% CI), *p*
	Reference	Reference	Reference
Cardiovascular Diseases ^1^			
Arterial stenosis	1.021 (0.841, 1.240), 0.8319	0.825 (0.673, 1.011), 0.0642	0.806 (0.653, 0.996), 0.0462
CAC	1.226 (0.891, 1.687), 0.2100	1.033 (0.734, 1.453), 0.8527	1.084 (0.763, 1.539), 0.6526
Metabolic Diseases			
Obesity	0.964 (0.851, 1.091), 0.5584	0.788 (0.693, 0.896), 0.0003	0.747 (0.649, 0.860), <0.0001
Hypertension	1.275 (1.157, 1.405), <0.0001	1.025 (0.918, 1.143), 0.6614	1.111 (0.986, 1.252), 0.0847
Diabetes	1.137 (0.951, 1.358), 0.1589	0.993 (0.820, 1.203), 0.9463	1.012 (0.830, 1.234), 0.9060
Metabolic syndrome	0.898 (0.812, 0.993), 0.0356	0.764 (0.685, 0.852), <0.0001	0.784 (0.680, 0.904), 0.0008
Other Diseases			
NAFLD *	1.147 (1.046, 1.257), 0.0034	0.829 (0.749, 0.917), 0.0003	0.940 (0.829, 1.064), 0.3265
CKD	0.795 (0.502, 1.260), 0.3293	0.851 (0.526, 1.376), 0.5096	0.837 (0.508, 1.381), 0.4864

Notes: “Reference” indicates that the nonhyperbilirubinemia group was the reference category (OR = 1) for comparison. CAC, Coronary artery calcification; NAFLD, non-alcoholic fatty liver disease; CKD, chronic kidney disease. Model 1: no covariates were adjusted. Model 2: age and sex were adjusted. Model 3: All diseases were adjusted for age, sex, smoking status, alcohol consumption, physical activity, ALT, GGT, and UA. Additional adjustments were made as follows: hypertension (BMI, diabetes, sCr); obesity (diabetes, hypertension, sCr); diabetes (BMI, hypertension, sCr); CKD (BMI, diabetes, hypertension); and arterial stenosis, CAC, metabolic syndrome, and NAFLD (BMI, diabetes, hypertension, sCr). ^1^ Analysis for cardiovascular diseases was performed using data from our cardiovascular cohort, whereas analyses for metabolic and other diseases were performed using our baseline cohort. * NAFLD is the variable after multiple imputation, with an original missing data rate of 13.8%.

## Data Availability

The data presented in this study are available on request from the corresponding author due to privacy.

## Abbreviations

The following abbreviations are used in this manuscript:

STB	Serum total bilirubin
TC	Total cholesterol
TG	Triglycerides
LDL-C	Low-density lipoprotein cholesterol
HDL-C	High-density lipoprotein cholesterol
VLDL	Very low-density lipoprotein
BMI	Body mass index
CAC	Coronary artery calcification
NAFLD	Non-alcoholic fatty liver disease
eGFR	Estimated glomerular filtration rate
